# One Health, Many Gaps: Rethinking Epidemic Intelligence in Resource-Limited Settings to Prepare for the Global Threat of Disease X

**DOI:** 10.3390/microorganisms13112615

**Published:** 2025-11-18

**Authors:** Blondy Kayembe-Mulumba, Anderson Kouabenan N’gattia, Marie Roseline Darnycka Belizaire

**Affiliations:** 1 Bordeaux Population Health Research Center, BPH, INSERM, U1219, University of Bordeaux, F-33000 Bordeaux, France; 2 National Institute of Public Hygiene, Ministry of Health, Abidjan 01 BPV 14, Côte d’Ivoire; nka1706@gmail.com; 3 World Health Organization, Country Office of the Central African Republic, Bangui BP 1416, Central African Republic; 4 School of Medicine, University of Alcalá, 28871 Madrid, Spain

**Keywords:** Disease X, pandemic preparedness, global threats, WHO priority diseases, health surveillance, one health, resource-limited settings, low- and middle-income countries

## Abstract

The emergence of high-threat pathogens—such as Ebola, Lassa fever, and most recently SARS-CoV-2—has highlighted critical weaknesses in global surveillance systems, particularly in resource-limited settings where many zoonotic spillovers originate. Despite the World Health Organization’s (WHO) prioritization of these diseases for research and development (R&D), the current surveillance infrastructures in these regions remain under-resourced, fragmented, and often reactive rather than anticipatory. This narrative review explored the literature and structured relevant findings in three key dimensions: (i) the structural and operational limitations of existing surveillance systems for the WHO priority diseases in resource-limited settings including challenges in data integration, laboratory capacity, workforce, and community engagement; (ii) how these surveillance gaps could delay detection and hinder the response to future emerging threats, particularly a hypothetical but inevitable Disease X; and (iii) innovative and context-adapted strategies to strengthen epidemic intelligence including integrated One Health surveillance, digital and genomic tools, participatory approaches, and regional data-sharing mechanisms. We argue that building agile, equity-centered, and decentralized surveillance systems is not only essential for managing known threats, but also foundational to the early detection and rapid containment of the next public health emergency in resource-limited settings. This review uniquely frames surveillance limitations in resource-limited settings as a global security concern and outlines context-adapted, equity-centered innovations to strengthen epidemic intelligence in preparation for Disease X.

## 1. Introduction

**Figure 1:**
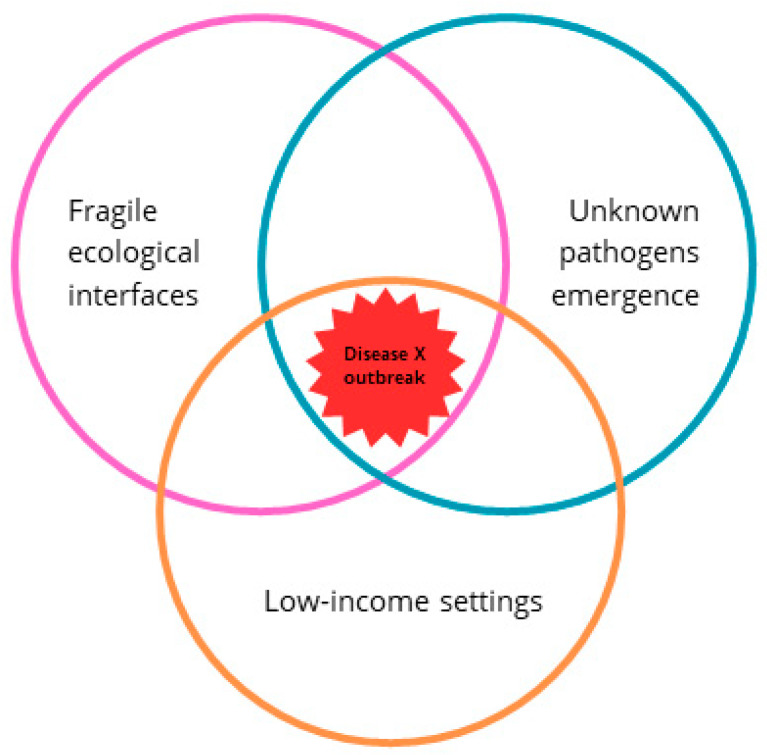
Disease X outbreak resulting from an overlap between fragile ecological interfaces, the emergence of unknown pathogens, and resource-limited settings. Framework designed by the authors. Pink circle indicates environmental gaps; Blue circle indicates surveillance gaps; Orange circle indicates structural and governance gaps.

The increasing frequency and severity of zoonotic disease outbreaks—such as Ebola virus disease (EVD), Lassa fever, and most recently, coronavirus disease 2019 (COVID-19)—have exposed significant gaps in global preparedness and highlighted the central role of early detection systems in mitigating public health crises [1,2]. These pathogens, many of which are listed under the World Health Organization (WHO) Research and Development (R&D) Blueprint for priority diseases, are often endemic in low- and middle-income countries (LMICs) where health systems are under-resourced and fragmented [3]. In these settings, surveillance systems frequently lack the financial investment, trained workforce, laboratory infrastructure, and digital tools required for timely detection, reporting, and response [4,5]. The consequences of weak surveillance extend far beyond national borders. Delayed identification of novel or re-emerging pathogens increases the risk of international spread, as seen in the 2014–2016 West African EVD outbreak and again with SARS-CoV-2. A pathogen with pandemic potential, especially if initially silent or misdiagnosed, can exploit the vulnerabilities in underfunded surveillance systems and remain undetected until its transmission footprint becomes unmanageable [6]. This scenario forms the conceptual basis of “Disease X”—an unknown pathogen with epidemic or pandemic potential that could emerge anywhere, but especially in settings with limited epidemic intelligence [7]. Figure 1 illustrates how fragile ecological interfaces and weak surveillance systems in LMICs may converge to create ideal conditions for undetected pathogen emergence.

Although the WHO’s prioritization efforts aim to stimulate research and preparedness for known high-risk pathogens, they do not fully address the foundational weaknesses in surveillance infrastructures in LMICs [8,9]. These include limitations in community-based reporting, laboratory confirmation capacity, integration of animal and environmental health surveillance, and data interoperability [10]. Furthermore, socioeconomic, political, and geographic barriers often hinder real-time information flow, resulting in fragmented data that delay coordinated action [11]. This narrative review thus aimed to provide a comprehensive analysis of these surveillance limitations in resource-limited settings. It further examined how such gaps heighten vulnerability to Disease X and hinder the global community’s ability to detect and respond to emerging pathogens. Finally, we propose a set of innovative and context-adaptable surveillance strategies—including genomic epidemiology, digital health platforms, and participatory approaches—to enhance the early warning and outbreak response capacities, aligned with the One Health framework. Unlike prior One Health reviews that emphasize conceptual alignment [12,13,14], our narrative highlights how fragmented surveillance infrastructures in resource-limited settings represent both a local vulnerability and a global blind spot for emerging pandemic threats. By connecting structural constraints to practical innovations, we offer a road map toward operationalizing equitable and anticipatory epidemic intelligence.

## 2. Materials and Methods

This narrative review was conducted to synthesize existing evidence and conceptual insights on epidemic intelligence systems in resource-limited settings, with a particular focus on their capacity to detect and respond to emerging threats such as Disease X within a One Health framework. The objective was to critically examine structural gaps, institutional weaknesses, and opportunities for integration across human, animal, and environmental health surveillance systems.

A structured approach was applied through March 2025 to gather diverse forms of data and literature from both academic and grey sources. We focused on publications from January 2005 to March 2025 to capture developments in surveillance systems and pandemic preparedness before and after major epidemics including SARS, H1N1, Ebola, and COVID-19. Sources included peer-reviewed journal articles, global and regional health agency reports (e.g., WHO, Food and Agriculture Organization [FAO], Africa Centers for Diseases Control and prevention [Africa CDC], etc.), technical working papers, evaluation briefs, and media reports. Scientific databases including Medline (PubMed), Scopus, and Web of Science were queried using combinations of the following keywords: “epidemic intelligence”, “One Health surveillance”, “zoonotic threats”, “low-income countries”, “resource-limited settings”, “Disease X”, and “pandemic preparedness”. Boolean operators were used to refine the search results (e.g., “One Health” AND “surveillance” AND (“low-income*” OR “resource-limited*”)). Additionally, national health strategies, integrated disease surveillance and response (IDSR) evaluations, and joint external evaluation (JEE) reports were consulted for country-level data on the surveillance capacities. Documents were included if they provided empirical data or conceptual analysis on early warning systems, cross-sectoral coordination, surveillance financing, or the integration of human, animal, and environmental data in LMICs. The review also mapped regional and international initiatives (e.g., Africa CDC Pathogen Genomics Initiative [PGI], REDISSE, WHO Hub for Pandemic and Epidemic Intelligence) to assess alignment with the needs of LMICs.

References were managed using Zotero software (version 6.0.36). Articles were screened manually for relevance based on titles and abstracts, and thematic extraction was conducted by consensus across authors following a narrative synthesis approach. Data extraction and thematic synthesis focused on five dimensions of epidemic intelligence: human health surveillance, animal health surveillance, environmental surveillance, genomic sequencing, and community-based event surveillance.

The purpose of this narrative review is not to offer a systematic or meta-analytical assessment but to contextualize epidemic intelligence within broader structural determinants of health system resilience. The findings are organized thematically to highlight key gaps, regional variability, and strategic opportunities to strengthen pandemic preparedness under the One Health paradigm and are structured in three sections. Section 3 portrays a landscape of surveillance systems gaps for priority diseases in resource-limited settings. Section 4 gleans potential implications of such surveillance gaps for future emerging threats from Disease X. In Section 5, we gather existing and emerging surveillance strategies leverageable in resource-limited settings in the preparedness for a Disease X outbreak. Section 6 summarizes the key recommendations for global health actors to foster an equitable surveillance framework for global security.

## 3. Surveillance Systems for WHO Priority Diseases: Current Landscape in Resource-Limited Settings

Despite global efforts to prioritize surveillance for epidemic-prone diseases, surveillance systems in resource-limited settings remain chronically under-resourced and fragmented. Table 1 provides a summary of critical gaps in epidemic intelligence systems in LMICs, their relevance to the emergence of Disease X, and real-world examples illustrating missed opportunities in early detection and response. This section examines key structural and operational limitations that impair the effective detection and response to WHO-listed priority diseases in these contexts.

### 3.1. Fragmented Health Information Systems

Many LMICs operate vertical, disease-specific surveillance programs, often supported by separate donor funding streams or international initiatives. For instance, parallel systems exist for HIV, tuberculosis, malaria, and vaccine-preventable diseases, often with dedicated reporting tools and limited interoperability [15]. This verticalization leads to the duplication of effort and hinders IDSR. Furthermore, cross-border data exchange is rare, and integration with One Health sectors—such as veterinary and environmental health—is minimal. Without harmonized health information platforms, critical signals for emerging outbreaks are missed or delayed, limiting the potential for the early detection of multi-system or zoonotic events [16]. Although tools such as JEE and IDSR frameworks offer strategic insights into surveillance capacity, their translation into operational improvements remains uneven. Many JEE recommendations remain aspirational due to limited domestic funding, a lack of policy continuity, and inadequate monitoring of the implementation outcomes.

### 3.2. Inadequate Laboratory and Diagnostic Capacity

Laboratory capacity is a critical component of surveillance, but remains weak in many LMICs. In the 2014–2016 West African EVD outbreak, laboratory bottlenecks were identified as a major driver of delayed response, with the confirmation of suspected cases often taking days or weeks [17]. Similarly, during the COVID-19 pandemic, access to real-time PCR (polymerase chain reaction), antigen testing, and genomic sequencing was limited across much of sub-Saharan Africa and South America, impeding the timely characterization of variants of concern [18]. For example, some countries—such as Benin and Bolivia—have had to repurpose GeneXpert (Sunnyvale, CA, USA) machines used for tuberculosis to test for COVID-19. As of 2022, fewer than 15 African countries had established routine genomic surveillance platforms, despite the emergence of SARS-CoV-2 variants such as Beta and Omicron from the continent [4]. Table 2 provides an overview of the current landscape of genomic surveillance in Africa, highlighting regional capacity inequalities and key initiatives aimed at enhancing pathogen genomics [19,20]. In addition to equipment and personnel gaps, an unreliable electricity supply presents a major obstacle to sustaining sequencing and diagnostic operations. Intermittent power can disrupt PCR machines, damage reagents, and interrupt digital data flows.

### 3.3. Workforce and Infrastructure Constraints

Surveillance is inherently labor-intensive, requiring a trained workforce for case investigation, data analysis, and laboratory testing at the local, national, and regional levels. However, most LMICs suffer from chronic human resource shortages due to underinvestment in public health training, poor retention, and the emigration of skilled professionals [21]. Until 2022, many African countries such as the Central African Republic, Mauritania and Zambia still exhibited an alarming density of community health workers (less than five per 10,000 population)—yet a critical asset of health surveillance. Infrastructure and equipment deficits compound this issue: unreliable electricity, poor Internet connectivity, and a lack of digital tools hinder timely reporting from peripheral facilities to central health authorities [22]. Financial resources are also limited with underpaid staff, making it difficult to implement the real-time surveillance and diagnosis of Disease X. Another factor to take into account is the multiplicity of institutions in charge of surveillance and biological diagnosis, and the “war” they wage against each other [23]. The synergistic effect of an inadequate health workforce, the lack of healthcare funding, corruption, weak regulatory bodies, and ineffective health governance policies are major challenges that preclude the achievement of UHC, and ultimately integrated health surveillance and security.

### 3.4. Underutilization of Community-Based Surveillance

Community-based surveillance (CBS) has shown success in previous emergencies, particularly in remote or underserved areas. During the West African EVD outbreak, CBS networks were instrumental in identifying clusters of unexplained deaths and alerting health authorities [24]. Despite its potential, CBS remains inconsistently implemented and underfunded. Many national systems lack mechanisms for systematically training, supervising, and integrating community health workers or volunteers into formal disease reporting systems [25]. Moreover, there is a lack of an effective funding framework to sustain the motivation of CBS works through reasonable incentives. Additionally, there is a limited use of mobile-based tools that could empower community members to participate in the real-time reporting of unusual health events. Collectively, these gaps create critical blind spots in the detection of priority infectious diseases. Strengthening the interoperability, laboratory capacity, human resources, and community engagement in surveillance systems is essential to prevent future pandemics and respond effectively to emerging threats including Disease X.

## 4. Implications for Future Emerging Threats: Disease X

The concept of Disease X was coined by the WHO to designate an unknown pathogen with pandemic potential, which could emerge from zoonotic reservoirs and rapidly spread across borders [26]. Though hypothetical in name, Disease X is grounded in the empirical reality that unknown viral and bacterial agents continue to emerge—often from fragile ecological interfaces where animal–human interactions are most intense. These zones of emergence disproportionately overlap with resource-limited settings, where high biodiversity, population mobility, and health system fragmentation create ideal conditions for undetected spillover events (Figure 1).

### 4.1. LMICs as Potential Epicenters of Disease X

LMICs, particularly in tropical regions, represent both the frontlines of emergence and the blind spots of global surveillance. The confluence of high biodiversity, weak veterinary public health systems, deforestation, and unplanned urban expansion near wildlife habitats intensifies the risk of zoonotic spillover [27]. In the Congo Basin and parts of West Africa, for instance, bush meat hunting and encroachment into forested areas are frequent, but the routine pathogen screening of wildlife is almost nonexistent due to technical, financial, and regulatory gaps [28]. Moreover, urbanization in these contexts often proceeds without commensurate investments in sanitation, housing, or healthcare infrastructure. Informal settlements, densely populated and poorly served by public health services, can serve as amplification hubs for a novel pathogen—especially if it is airborne, has asymptomatic transmission, or a long incubation period [29]. While these issues are mainly discussed in the African context, as a high-risk region for zoonotic spillovers and systemic surveillance gaps, the implications are global. Strengthening surveillance in LMICs should not be seen as aid, but as planetary health security investment—reinforcing both endemic disease control and the early detection of emerging pandemics.

### 4.2. Peripheral Surveillance: The First Line of Defense

Emerging pathogens rarely originate in tertiary hospitals or reference labs; instead, they appear as unusual clinical syndromes at the periphery—rural clinics, community health posts, or informal care settings. However, surveillance systems in LMICs remain highly centralized, focusing on notifiable diseases with limited capacity for event-based, syndromic, or genomic surveillance at the community level [30]. During the COVID-19 pandemic, early signs of community transmission in some African and South Asian countries went unrecognized due to poor diagnostic access, a lack of real-time data systems, and the absence of genomic monitoring [4,31]. These blind spots delayed detection and undercut mitigation efforts, allowing variants of concern to spread undetected until they reached international airports or hospitals. The same asymmetries will persist—and possibly worsen—during Disease X unless proactive, decentralized surveillance strategies, such as the 7-1-7 target [32], are implemented without further delay.

### 4.3. Genomic Surveillance and Global Equity

Genomic sequencing, a cornerstone of pandemic preparedness, remains deeply unequal. In 2021, over 90% of submitted SARS-CoV-2 sequences came from high-income countries (HICs), even though many LMICs had significant outbreaks [33]. The gap was driven by disparities in infrastructure (e.g., PCR labs, sequencers), skilled workforce, reagent procurement, and bioinformatics capacity. This surveillance inequity has serious implications for the global management of Disease X, especially if the pathogen evolves rapidly or escapes diagnostic detection. As presented in Table 1, programs like the Africa CDC’s PGI and WHO’s BioHub offer hope but are still in early development and are underfunded relative to need [19]. Without investments in regional sequencing hubs, portable sequencers (e.g., MinION [Oxford, UK]), and data-sharing platforms, LMICs will continue to depend on external support and delayed outbreak intelligence. Furthermore, data sovereignty concerns and a lack of equitable benefit sharing have made pathogen sharing politically sensitive. The precedent set by Indonesia in withholding H5N1 samples over vaccine access concerns underscores the need for transparent, reciprocal agreements on the use of genomic and epidemiological data from LMICs [34].

### 4.4. One Health Blind Spots

The emergence of Disease X is almost certainly linked to human–animal–environment interactions, however, One Health surveillance—the integration of human, animal, and environmental health data—remains nascent in most LMICs. Veterinary surveillance is under-resourced, with limited capacity for the active surveillance of livestock, wild animal populations, or wet markets [35]. Furthermore, environmental drivers like land-use change, climate variability, and pollution are poorly monitored or disconnected from health data systems. For example, deforestation data from satellite imagery or wildlife migration patterns are rarely integrated into outbreak forecasting models or health sector decision-making [36]. Disease X preparedness must therefore extend beyond the ministries of health to include environmental agencies, forestry departments, and urban planners, embracing a genuinely intersectoral epidemic intelligence framework.

### 4.5. From Detection to Action: The Political Economy of Epidemic Intelligence

Surveillance without response capacity can breed disillusionment. For Disease X, timely detection must be matched by timely action—isolation protocols, clinical care pathways, contact tracing, and risk communication. However, in many LMICs, surveillance data are not integrated into decision-making loops or are siloed in parallel systems run by NGOs (non-government organizations), academic institutions, or donors [37]. Moreover, the political economy of surveillance is often skewed toward external visibility (e.g., meeting IHR obligations) rather than internal utility (e.g., local outbreak response). Despite their high citation in global health planning, evaluations such as JEEs often have limited influence on the day-to-day operations in LMICs. Fragmented follow-up, lack of accountability mechanisms, and top-down donor-driven priorities dilute their local impact. The COVID-19 experience revealed that some countries hesitated to report new variants due to the fear of travel bans or reputational damage, thereby discouraging transparency [38]. To overcome these barriers, epidemic intelligence systems must be trusted, timely, and actionable. Community participation, feedback loops, and data democratization (e.g., public dashboards) can help build legitimacy and public trust in outbreak alerts—critical for controlling high-stakes threats like Disease X.

### 4.6. Lessons from Mpox and COVID-19

The Mpox epidemic in 2022–2024, largely driven by Clade I in Central Africa, illustrated how neglected surveillance priorities can allow pathogens to circulate undetected for years before global attention is triggered by cases in HICs. Similarly, the global failure to prioritize COVID-19 diagnostic equity and vaccine access revealed how resource gaps translate into epidemiological risks for all. For Disease X, these lessons must inform a new generation of epidemic intelligence systems grounded in equity, local capacity, and intersectoral collaboration. The next outbreak may not wait for new infrastructure or policies—it will emerge through the cracks of our current systems. Closing those cracks (gaps) must begin no later than now.

## 5. Rethinking Surveillance: Emerging Strategies for Resource-Limited Settings

To close surveillance gaps and mitigate the risk of emerging epidemics, including Disease X, LMICs must transition from reactive, fragmented surveillance systems to proactive, integrated, and technologically enabled approaches. This section highlights five key innovations showing promise in strengthening epidemic intelligence in resource-constrained contexts.

### 5.1. Integrated One Health Surveillance

A growing body of evidence supports the importance of a One Health surveillance approach—one that simultaneously monitors human, animal, and environmental health sectors—to detect and preempt zoonotic spillovers [39]. This is particularly relevant as over 60% of emerging infectious diseases are of animal origin, with many first identified in regions with high human–animal interface and weak surveillance [1]. Uganda and Ghana have demonstrated the feasibility of integrated One Health surveillance systems through the use of shared reporting platforms, joint outbreak investigations, and regular cross-sectoral coordination meetings [40]. For example, Uganda’s National One Health Platform facilitated real-time data exchange between veterinary and public health services during zoonotic disease threats such as Rift Valley fever and anthrax [41,42]. Despite these successes, scaling such models requires harmonized policy frameworks, interoperable digital systems, and sustained funding mechanisms [41].

### 5.2. Genomic Surveillance and Digital Platforms

Genomic surveillance provides critical insights into pathogen evolution, transmission chains, and vaccine escape mutations. The COVID-19 pandemic highlighted the transformative power of real-time genomic data but also revealed stark inequities in sequencing capacity. As of 2020, less than 2% of global SARS-CoV-2 sequences originated from sub-Saharan Africa [33]. To address this, the Africa CDC launched the Africa PGI, a USD 100 million effort to expand sequencing and bioinformatics infrastructure across 20+ countries [19]. In Nigeria, the National Center for Disease Control successfully integrated genomics into its national COVID-19 response, tracking introductions and variants using platforms such as GISAID (Munich, Germany) and Nextstrain (Seattle, WA, USA) [43]. However, challenges persist including limited trained personnel, inconsistent reagent supply, and the absence of legal frameworks for data sharing.

Beyond genomics, digital surveillance tools like the District Health Information System 2 (DHIS2) and the Surveillance Outbreak Response Management and Analysis System (SORMAS) have gained traction across Africa. DHIS2 is now used in over 40 African countries for routine health data collection, while SORMAS enables real-time contact tracing and case reporting [44]. In Ghana, SORMAS was instrumental in managing both the COVID-19 and Lassa fever outbreaks, demonstrating the platform’s versatility and scalability [45,46]. A full implementation of IDSR, under the 7-1-7 approach, can strengthen and integrate different surveillance strategies including indicator-based surveillance [32].

While platforms such as GISAID and Nextstrain have democratized genomic surveillance, their utility in LMICs remains constrained by unequal Internet access, limited computational capacity, and reliance on subscription-based analytical tools. Many LMIC institutions lack licenses for bioinformatics software, and often face delays in accessing high-impact surveillance publications due to paywalls. This inequity in data access and use undermines timely decision-making and exacerbates North–South asymmetries in epidemic response. Additionally, concerns about data sovereignty and benefit sharing deter open data contributions from LMICs, as seen in past cases involving H5N1 and SARS-CoV-2. Furthermore, to address chronic power outages affecting laboratory reliability and digital storage, some countries have begun to adopt solar-powered mini-grids, battery-backed data centers, and cloud-based redundancy systems hosted in more stable regional hubs. Strengthening public–private partnerships in energy and digital infrastructure is essential to protect data integrity and ensure uninterrupted genomic surveillance operations.

### 5.3. Community-Based and Participatory Approaches

CBS is a cornerstone of early detection in remote or underserved settings. It empowers local actors—community health workers, traditional healers, and volunteers—to report unusual events, often before cases reach formal health facilities. CBS is particularly effective for detecting diseases with social stigma or when formal surveillance systems are overwhelmed. The WHO’s IDSR strategy has increasingly emphasized CBS integration. In Sierra Leone, CBS was pivotal during the 2014–2016 EVD outbreak, with trained community informants submitting weekly alerts via mobile phones [24]. Evaluations from Uganda and South Sudan show that CBS can lead to timelier outbreak alerts and strengthen community trust in public health responses [47]. However, CBS programs often suffer from inadequate training, poor supervision, and a lack of incentives. Sustainable CBS systems require continuous investment, integration into formal health information systems, and the use of digital tools to streamline reporting and feedback [25]. Participatory surveillance platforms like AfyaData and Argus are also emerging, enabling communities to submit real-time reports via SMS or smartphone applications [48]. Another approach to making surveillance more effective is to set up a responsible community surveillance system. Leveraging the experience from the ESSENCE network in the United States, a multimedia application from the Marine Corps, accessible to the population, will enable voluntary notification, via all digital media, of any health event detected [49].

Social insights are evenly central to implementing CBS and response measures. As He et al. [50] emphasize, public trust, risk perception, and sociocultural beliefs significantly influence outbreak reporting, adherence to public health guidance, and vaccine acceptance. In resource-limited settings, misinformation or historical mistrust of the authorities has undermined compliance with disease control efforts. Therefore, integrating social science tools—such as community dialogue, participatory ethnography, and behavioral surveillance—into epidemic intelligence systems is critical for translating early warning into effective action.

### 5.4. Regional Collaboration and Data Sharing

Infectious diseases do not respect borders, making regional collaboration a critical component of effective surveillance. Initiatives such as the West African Health Organization (WAHO) and the East, Central, and Southern Africa Health Community (ECSA-HC) promote harmonized surveillance standards, data sharing, and joint epidemic response among member states. The Africa CDC’s establishment of five Regional Collaborating Centers (RCCs) has further strengthened continent-wide coordination. These RCCs support member states through technical assistance, cross-border outbreak investigations, and capacity-building workshops [51]. For example, during the 2018 EVD outbreak in the Democratic Republic of Congo, RCCs facilitated surveillance support and laboratory training for neighboring countries including Rwanda and Uganda. However, effective regional surveillance still faces barriers such as data ownership concerns, weak governance structures, and a lack of sustainable financing. To move forward, regional organizations must establish binding agreements on data sharing, strengthen public health legal frameworks, and invest in shared digital platforms like the African Surveillance Informatics Platform [52].

Beyond regional coordination, pandemic preparedness must be institutionalized through binding cross-border surveillance agreements and joint simulation exercises. The East African Community and ECOWAS have made strides in this direction, but the harmonization of surveillance thresholds, data formats, and legal obligations remains limited. The COVID-19 pandemic showed the need for shared protocols on sample transport, outbreak notification, and border health measures to prevent the fragmentation of response efforts. It is hoped that the recent WHO Pandemic Agreement will be the real catalyst needed for achieving the expected global health equity and preparedness [53].

### 5.5. Leveraging Non-Traditional Data Sources

The COVID-19 pandemic demonstrated the utility of non-traditional data sources—such as mobile phone location data, social media trends, and Internet search patterns—in monitoring population movement and detecting early outbreak signals. These data sources can complement traditional surveillance, especially where formal systems are weak or delayed. In Kenya and South Africa, mobility data derived from mobile phone networks were used to track adherence to movement restrictions and predict geographic hotspots of transmission [54]. Similarly, Google Trends and Twitter data have been used to detect influenza-like illness trends in Nigeria and Uganda, often correlating with official surveillance indicators [55,56,57]. Additionally, the Epidemic Intelligence from Open Sources (EIOS) is a global resource that can assist health authorities in public health decision-making [58]. Syndromic surveillance systems like HealthMap and ProMED aggregate informal reports to provide early warnings of potential outbreaks globally. While promising, the use of non-traditional data sources raises ethical and technical concerns including data privacy, representativeness, and the risk of misinformation. Robust governance frameworks and public–private partnerships are needed to ensure that these tools are used responsibly and effectively [59,60,61].

## 6. Recommendations and Way Forward

To avert future pandemics and minimize the risk of a novel Disease X emerging undetected, the global health community must prioritize sustainable investments in surveillance systems tailored to the needs of resource-limited settings. The current surveillance systems must be transformed to be inclusive, anticipatory, and adaptable. Strengthening surveillance in LMICs is not only a local imperative, but also a global necessity. Only through coordinated local, national, regional, and global efforts can we effectively preempt the emergence and international spread of the next Disease X. Based on the evidence presented, we recommend the five actions in Box 1 to prepare for the potential Disease X pandemic. These include investing in integrated, decentralized surveillance systems; strengthening laboratory networks and genomic sequencing; enhancing data interoperability and governance; building and retaining a skilled public health workforce; and fostering equitable international partnerships.

Box 1 **Five recommended actions to prepare LMICs for the potential Disease X pandemic.**

**1. Investing in integrated, decentralized surveillance systems:** Health systems must move beyond vertical programs and embrace integrated approaches that link CBS, facility-level data, and One Health intelligence. Embedding CBS into formal structures and leveraging digital tools will improve early detection at the community level and facilitate rapid response.

* * 

**2. Strengthening laboratory networks and genomic sequencing:** Sustained financing, regional reference labs, and technology transfer are essential to expand diagnostic and sequencing capacity. Legal frameworks for safe data sharing and bio-sample governance should accompany these investments.

* * 

**3. Enhancing data interoperability and governance:** Real-time, cross-sectoral data sharing across human, animal, and environmental health sectors requires interoperable digital systems and robust information governance mechanisms that respect privacy and promote transparency.

* * 

**4. Building and retaining a skilled public health workforce:** National and regional training hubs should be supported to produce a cadre of epidemiologists, laboratorians, and computer scientists. Retention strategies must address working conditions, career development, and equitable remuneration.

* * 

**5. Fostering equitable international partnerships:** Global initiatives must prioritize local ownership, align with national priorities, and strengthen public sector capacity, as supported by the recently concluded WHO Pandemic Agreement. Donor funding should shift from short-term project cycles to long-term systems strengthening.

## 7. Conclusions

LMICs remain at the epicenter of many emerging infectious disease threats, but their surveillance systems are often fragmented, under-resourced, and poorly integrated. These systemic vulnerabilities hinder timely detection, response, and containment—exacerbating the risks posed by WHO-priority pathogens and increasing the likelihood that a novel pathogen, such as “Disease X”, could spread undetected. This review underscored the urgent need to shift from reactive, vertical disease surveillance toward integrated systems that are community-informed, technology-enabled, and grounded in the One Health framework. Successful models exist including genomic surveillance networks, community-based detection strategies, and regional collaboration mechanisms. However, these approaches remain unevenly adopted and inadequately funded in many low-resource settings. The path forward must include sustained investments in laboratory and digital infrastructure, workforce development, cross-sectoral data interoperability, and equitable international partnerships that prioritize local ownership and capacity strengthening. Moreover, political commitment is essential to institutionalize and scale these innovations beyond pilot phases. Addressing surveillance gaps in resource-limited settings is not only a matter of national health resilience—it is a global security priority. As pathogens increasingly cross borders and species, our collective preparedness depends on the strength of the most vulnerable links in the global surveillance chain. Strengthening these systems today is the surest way to prevent the emergence, amplification, and global dissemination of the next pandemic threat.

## Figures and Tables

**Table 1:** Surveillance system gaps and implications for emerging threats by surveillance domain in resource-limited settings.

Surveillance Domains	Common Gaps	Implications for Disease X Detection	Past Examples
Community-based surveillance	Lack of digital tools, undertrained health workers	Spillover may go undetected at the local level	Ebola in Guinea (2014)
Zoonotic/veterinary surveillance	Poor lab infrastructure, low funding, weak reporting	Missed early warning of animal–human transmission	Rift Valley fever in Sudan
Genomic sequencing	Low lab throughput, shortage of trained bioinformaticians	Inability to track pathogen mutations	COVID-19 variants in Africa
Environmental health surveillance	Weak coordination with health ministries	Poor outbreak prediction (e.g., vector-borne or climate-linked diseases)	Dengue in Southeast Asia
Event-based surveillance (EBS)	Informal, poorly institutionalized systems	Delayed outbreak recognition	Cholera in Haiti (2010)
Cross-sectoral data integration	Legal, bureaucratic, and IT system silos	Delayed coordinated response	Mpox in Africa

Abbreviations: COVID-19, Coronavirus disease 2019; IT, Information technology.

**Table 2:** Current landscape of genomic surveillance in African regions. Data adapted from the Africa CDC and Pasteur Institute [19,20].

African Regions	Countries with Functional NGS Capacity *	CoE †	Notable Initiatives/Networks
West Africa	10	3	AFROSCREEN project enhancing sequencing platforms in West and Central Africa ‡
East Africa	6	2	Africa PGI supporting regional sequencing hubs §
Central Africa	5	1	AFROSCREEN project bolstering genomic surveillance
North Africa	4	1	Morocco’s established sequencing infrastructure
Southern Africa	5	2	South Africa’s multiple institutions and training centers

Abbreviations: CoE, Center of Excellence; NGS, next-generation sequencing; PGI, Pathogen Genomics Initiative. * Functional NGS capacity: Refers to countries with operational next-generation sequencing platforms capable of contributing to genomic surveillance efforts. † Centers of Excellence: Designated institutions recognized for their advanced capabilities in genomic sequencing and analysis, serving as regional hubs. ‡ AFROSCREEN Project: A EUR 10 million initiative launched in 2021 to establish and strengthen sequencing platforms in West and Central Africa including Madagascar. § Africa PGI: The Africa Pathogen Genomics Initiative aims to expand sequencing platforms, build bioinformatics expertise, and promote regional data sharing across the continent.

## Data Availability

No new data were created or analyzed in this study. Data sharing is not applicable to this article.

## Abbreviations

The following abbreviations are used in this manuscript:

Africa CDC	Africa Centers for Diseases Control and prevention
CBS	Community-based surveillance
COVID-19	Coronavirus disease 2019
EVD	Ebola virus disease
IDSR	Integrated disease surveillance and response
LMICs	Low- and middle-income countries
PCR	Polymerase chain reaction
WHO	World Health Organization
